# Terahertz meta-chip switch based on C-ring coupling

**DOI:** 10.1515/nanoph-2021-0646

**Published:** 2022-01-04

**Authors:** Sen Gong, Hongxin Zeng, Qianyu Zhang, Chunyang Bi, Lan Wang, Tianchi Zhou, Ziqiang Yang, Yaxin Zhang, Fanzhong Meng, Zhenpeng Zhang, Yuan Fang

**Affiliations:** Sichuan Terahertz Communication Technology Engineering Research Center, School of Electronic Science and Engineering, University of Electronic Science and Technology of China, Chengdu, China; Yangtze Delta Region Institute (Huzhou), University of Electronic Science and Technology of China, Chengdu, China; National Key Laboratory of Application Specific Integrated Circuit, Hebei Semiconductor Research Institute Shijiazhuang 050051, Shijiazhuang, China

**Keywords:** C-ring, InP-HEMT, switch, terahertz meta-chip

## Abstract

Terahertz switch is one of the key components of future communication, radar, and imaging systems. Limited by the strong electromagnetic coupling in subwavelength scale, the traditional terahertz switch is difficult to meet the increasing application requirements. In this paper, a parallel topology terahertz meta-chip switch based on the combination of equivalent circuit theory and electromagnetic coupling is proposed. The meta-chip is realized by adjusting the density of two-dimensional electron gas of InP-HEMT, which converts the electromagnetic coupling between the microstructure and microstrips. By using the 90 nm gate length InP-HEMT process, a C-ring loaded meta-chip is fabricated and tested in this paper. The results show an insertion loss lower than 1 dB with a 10 dB switching ratio, which is 20% higher than that without C-ring while ensuring the rather low insertion loss. It shows that the presented mechanism has positive significance for the design of terahertz band functional devices.

## 1 Introduction

Terahertz has wide applications in the next-generation communication, radar and imaging systems because of its high frequency and wide bandwidth [1], [2], [3], [4]. The increasing demand of application promotes the rapid development of terahertz functional devices such as multiplier/mixer [5–7], modulator [8, 9], phase shifter [10, 11], and so on. Terahertz switch is one of the key devices to realize signal isolation in these systems, and has also become a research hotspot.

From the view point of traditional radio-frequency circuit, terahertz switch can be realized by using high-performance active materials such as InP, GaAs et al. and impedance matching technology [12], [13], [14], [15], [16]. For example, Thome F et al. realized a single-pole-double-throw (SPDT) switch operating at 122–330 GHz by four GaAs-HEMT, the average insertion loss and switching ratio was 2.2 and 17.4 dB, respectively [12]. Shivan T et al. presented another on-chip SPDT switch with six InP DHBTs, which operated at 220–325 GHz with an insertion loss lower than 5 dB, a switching ration larger than 30 dB [13]. However, limited by the performances of the active materials and the couplings at the sub-wavelength scale, there are great challenges to improve this kind of on-chip switch further in terahertz region.

Metamaterials [17], [18], [19], which consists of artificial microstructures, introduce a new orientation for designing electromagnetic functional devices by using the coupling from these microstructures [20], [21], [22], [23], [24], [25], [26], [27], [28], [29], [30], [31], [32], [33], [34], [35]. Depending on the “ON/OFF” characteristic of the active materials, the electromagnetic coupling of the microstructure is changed, corresponding to the modulation of the incident waves. For instance, a terahertz meta-switch based on split-ring resonators with GaAs loading was presented by H.-T. Chen et al. in 2006 [21]. Thanks to the development of metamaterials, more and more coupling mechanisms are employed to realize the meta-devices. In 2012, J. Wu et al. demonstrated an active electromagnetically induced transparency (EIT), which opened up the possibility for chip-scale ultrafast devices, such as meta-switches and meta-modulators [22]. In the same year, a photoinduced handedness switch in terahertz region was reported by S. Zhang et al. [23]. In 2019, an active control of terahertz was also realized by vanadium-dioxide-embedded metamaterials by C. Zhang et al. [24]. Except this, more basic characteristics of the metamaterials was designed to control electromagnetic waves, such as the bound states in the continuum, which was reported to be used in an all-dielectric active terahertz device by S. Han et al. [25]. However, there are still many challenges for the meta-switch to meet the rapidly growing demands. To obtain a promising switching ratio, many active materials are needed in a meta-switch to break the natural robustness from the periodical structure. This puts forward higher requirements for processing consistency and auxiliary feed circuit design. In addition, since the interaction between metamaterials and electromagnetic waves, meta-switch often operates at quasi-optical mode, and it is difficult to realize on-chip integration.

This paper presents a mechanism of parallel topology terahertz on-chip meta-switch circuit, which is named as meta-chip switch, based on the coupling of on-chip propagating waves between the microstrip branches, C-ring and InP-HEMTs. By adjusting the density of two-dimensional electron gas (TDEG) of the InP-HEMT, the impedance of the parallel topology meta-branch is adjusted by the on-chip coupling, resulting in the “ON/OFF” status of the meta-chip. Further, according to the revealed relation between the on-chip coupling and the meta-chip, the switch can be optimized by designing the microstructure on demand. Based on the revealed mechanism, a meta-chip switch with a single transistor working at 220 GHz is designed and fabricated. The results show an insertion loss lower than 1 dB with a 10 dB switching ratio, which is 20% higher than that without C-ring while ensuring the rather low insertion loss. It indicates that the presented mechanism is conducive to enhancing the efficiency of the transistors in the circuit by electromagnetic coupling, which has positive significance for terahertz communication, radar, and imaging systems in the future.

## 2 The theoretical analyses

### 2.1 The optimal switching of meta-chip

**Figure 1:**
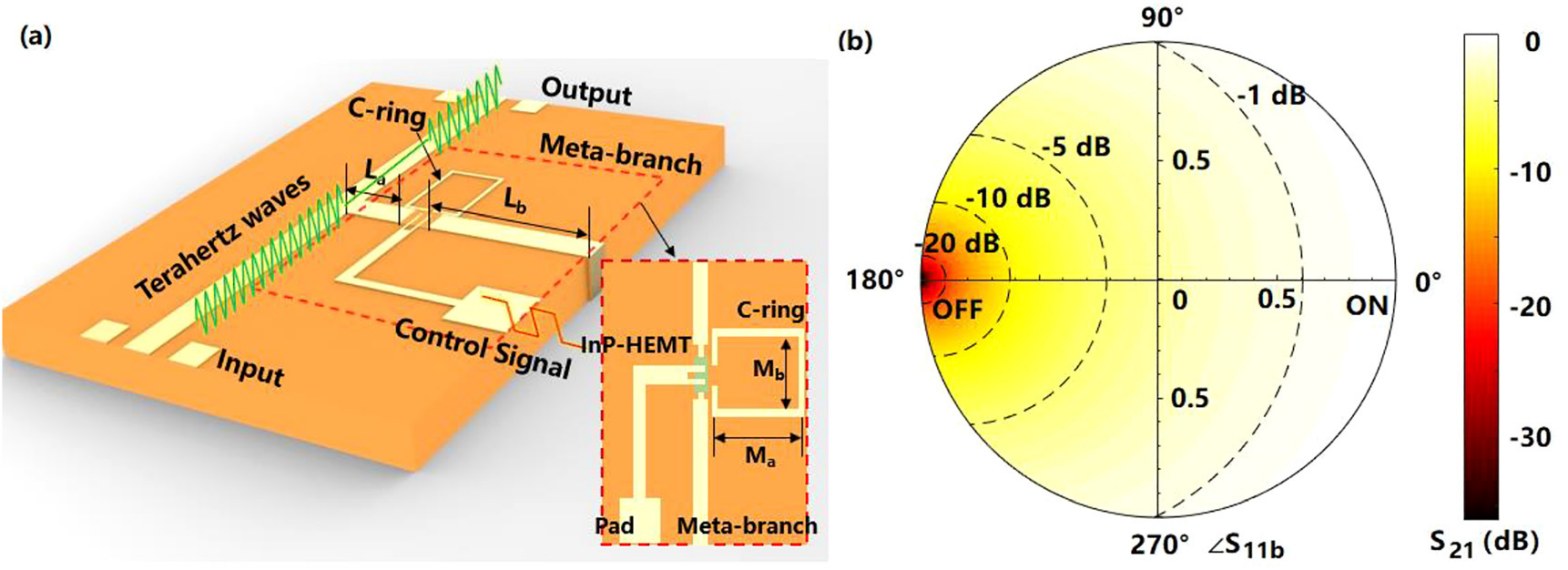
The shematic of the meta-chip and the optimal “ON/OFF” points. (a) The schematic of the meta-chip, and the meta-branch consists of microstrips, InP-HEMT and a C-ring; (b) The relation between the *S*_21_ of the meta-chip and the *S*_11*b*_ of the meta-branch/normal branch, in which all possible *S*_11*b*_ is described by the points in the unit cycle by polar coordinate, and the *S*_21_ is expressed by the gradually changed background color.

The schematic of the parallel topology meta-chip switch is shown in Figure 1a, in which a meta-branch is parallelly connected to the main microstrip. As shown in the inset of Figure 1a, the meta-branch consists of microstrip branches, InP-HEMT and C-ring, the InP-HEMT is embedded in the microstrip branch, and the C-ring is loaded on the one side of the HEMT. Compared with the traditional switching circuit without C-ring loading, the meta-chip switch is realized by the impedances converting induced by different on-chip couplings in the meta-branch.

Since the influences of the on-chip coupling are presented in the form of *S*_11*b*_ of the separated meta-branch, the dependences of the *S*_21_ of the meta-chip on all possible *S*_11*b*_ are studied first to clarify the physical process of the switch. First, considering the quasi-TEM mode in the microstrip lines and ignoring the coupling between the main microstrip line and the meta-branch, the relationship between the meta-branch and the meta-chip can be studied by the continuity of tangential electric field and conservation of electromagnetic energy. And then, for all the one branch parallel topology circuit with the same characteristic impedance, there is:

(1) $$S_{21}=2\frac{S_{11b}+1}{3+S_{11b}}$$

The calculated results of Eq. (1) are demonstrated in Figure 1b by polar coordinates, in which the background in gradually changed color indicates the absolute value of *S*_21_ at the corresponding *S*_11*b*_ in the complex unit-circle.

As shown in Figure 1b, the optimal “OFF” status is located at the point of *S*_11*b*_ with amplitude of 1 and phase of 180°. When the phase is 180°, a group of electric fields with equal amplitudes and opposite directions appears at the parallel port of the meta-branch, which indicates a 0 synthetic electric field. According to the continuity of tangential electric field, the electric field at the output port of the meta-chip is also 0, corresponding to the ideal “OFF” status. This conclusion is consistent with the equivalent circuit theory, because the 0 electric field at the port of the meta-branch also means 0 impedance. The ideal “ON” status at the point with amplitude of 1 and phase of 0° can also be explained by similar reasons. In this way, the C-ring can be designed according to the optimal “ON/OFF” points of the meta-chip.

### 2.2 The coupling in the meta-branch

The spectra of *S*_11*b*_ caused by the on-chip coupling between the C-ring and microstrip in the meta-branch is studied by simulation, in which the substrate is InP with the thickness of 70 μm, and the linewidth of microstrips is 48 μm. The lengths of two microstrip branches, which connect the InP-HEMT with the length of 50 μm in the meta-branch, are 100 and 270 μm respectively. The C-ring is loaded on one side of the InP-HEMT with a spacing of 2 μm, and the ring gap is 6 μm. The length of the upper and lower arms of the ring is 150 μm, the length of the left and right arms is 100 μm, and the ring linewidth is 6 μm.

**Figure 2:**
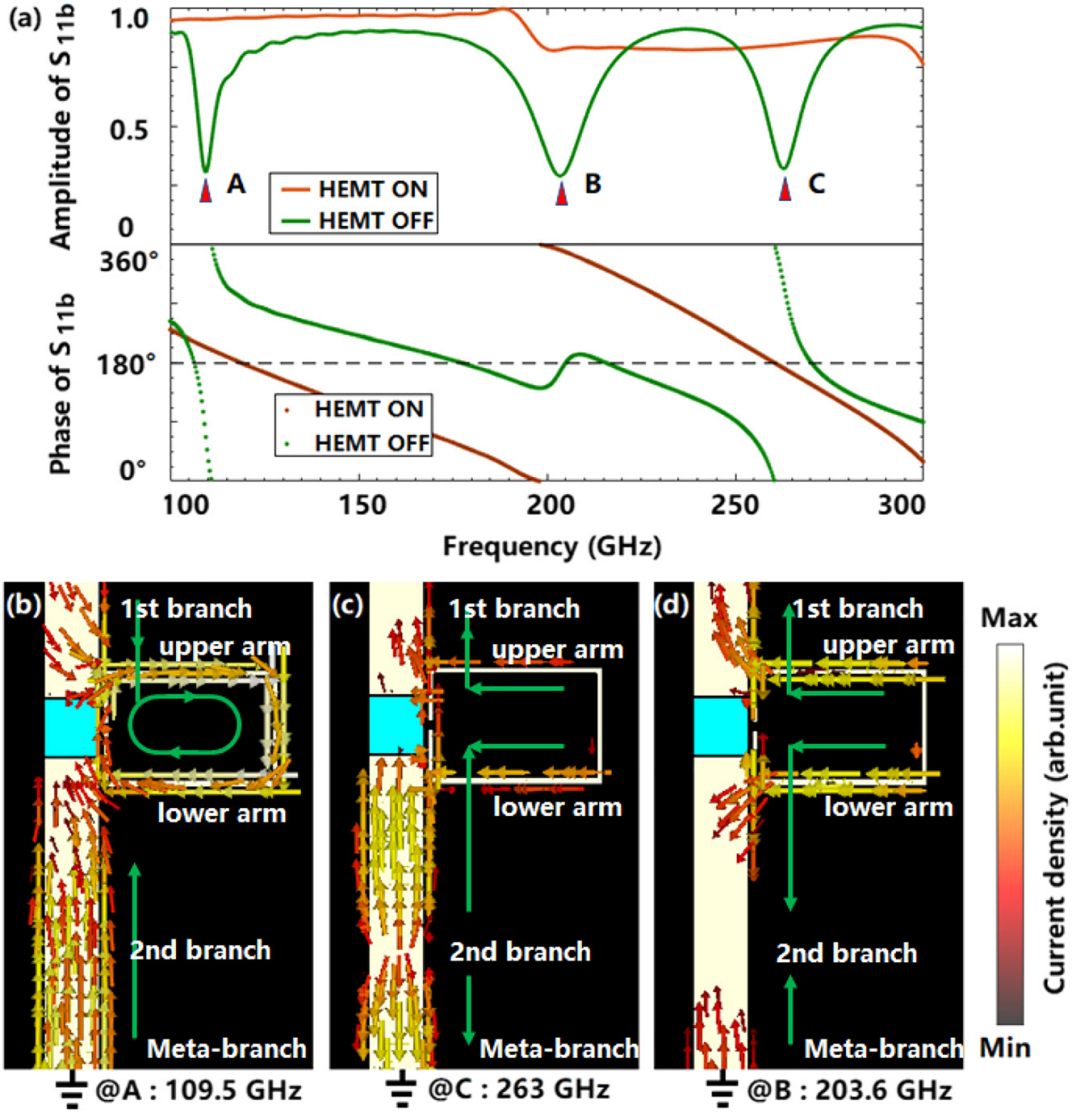
The spectra S_11b_ of meta-branch and the current distrbutions at the resonance points. (a) The spectra of the *S*_11*b*_ for the InP-HEMT with voltage applied; (b) the current distribution at peak A; (c) the current distribution at peak C; and (d) the current distribution at peak B.

When a voltage is applied to the InP-HEMT, the TDEG is exhausted, and the induced currents in the meta-branch is blocked. Thus, the induced charges are accumulated on the ends of the branches, which leads to a strong coupling between the C-ring and the branches. The amplitude and phase spectra of *S*_11*b*_ for the InP-HEMT with a voltage applied are demonstrated as green lines in Figure 2a. It is found that there appear three resonance peaks which are marked as A, B and C, respectively. And their current distributions are shown in Figure 2b–d, respectively. As shown in Figure 2b, the induced currents are mainly distributed on the second branch and C-ring for peak A. On the C-ring, an entire current loop is formed, which indicates an LC resonance. While on the second branch, a standing wave mode is caused by the ground boundary conditions. Thus, peak A is caused by the coupling between the LC resonance and standing wave mode. For the current distribution of peak C demonstrated in Figure 2c, the induced currents with the same phase on the upper and lower arms of the C-ring indicate a dipole resonance, which couples with the high order standing wave mode on the second branch. For the peak A and C are all mainly caused by the coupling between the second branch and C-ring, the fields on the first branch are affected indirectly by the coupling between the two branches though the InP-HEMT with exhausted TDEG. Accordingly, the coupling is equivalent to change the effective electric length of the branches, and then the *S*_11*b*_ of meta-chip shows similar phase characteristic as that of single microstrip branch without C-ring loading, as shown in Figure 2a. While for peak B, the resonance originates from the coupling between both the two branches and the C-ring, as shown in Figure 2d. It is found that the first branch and the upper arm, the second branch and the lower arm are connected as a whole, respectively, by the induced couplings. And then a dipole resonance is formed on the entire meta-branch. Accordingly, the phase characteristic of *S*_11*b*_ at peak B is kept around 180°, as the green line shown in Figure 2a.

When the voltage is removed, the TDEG recovers, there is no charge accumulation between the two branches in the meta-branch, and the field propagates as quasi-TEM mode. At this time, the weak coupling between the C-ring and the branch causes a perturbation to the field of the meta-branch, so that the amplitude and phase characteristics of *S*_11*b*_ are similar to that of a single microstrip branch, as the brown lines shown in Figure 2a.

### 2.3 The “ON-OFF” status of the meta-chip

**Figure 3:**
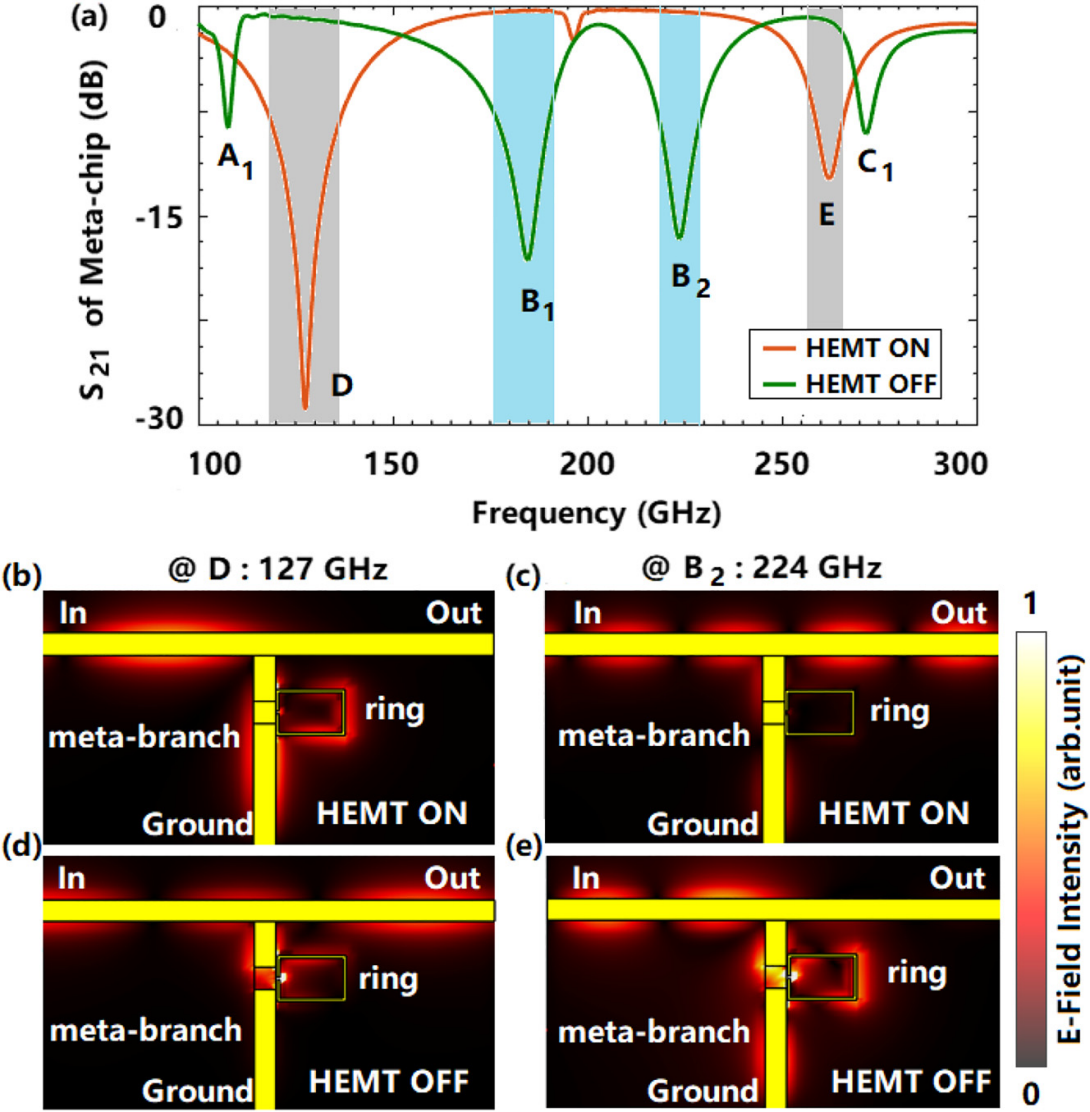
The spectra of S_21_ of meta-chip and the field contour maps at the “ON/OFF” points. (a) The amplitude spectra of the *S*_21_ of the meta-chip; (b) to (c) The electric fields distribution of the meta-chip at points D and B_2_, respectively.

Accordingly, the coupling mode in the meta-branch can be controlled by the TDEG of InP-HEMT, so as to realize the switching of the meta-chip depending on the relation revealed by Eq. (1). As the gray and blue regions shown in Figure 3a, according to the amplitude and phase characteristics of *S*_11*b*_ at peak B induced by strong coupling, two “OFF” status B_1_ and B_2_ of the meta-chip are formed. While for the peaks D and E of the meta-chip shown in the gray regions, the “OFF” status originates from the impendence converting owing to the propagation with perturbation induced by the weak coupling. The physical process can be further confirmed by the contour maps. It is found in Figure 3b and c, in the case of perturbation, the field propagates mainly as quasi-TEM mode, and no obvious resonance is formed on the C-ring. While in Figure 3d and e, the strong coupling causes resonance at the C-ring, and further changes the impedance of the meta-branch after transmission through the first microstrip to form the “ON/OFF” status of the meta-chip.

**Figure 4:**
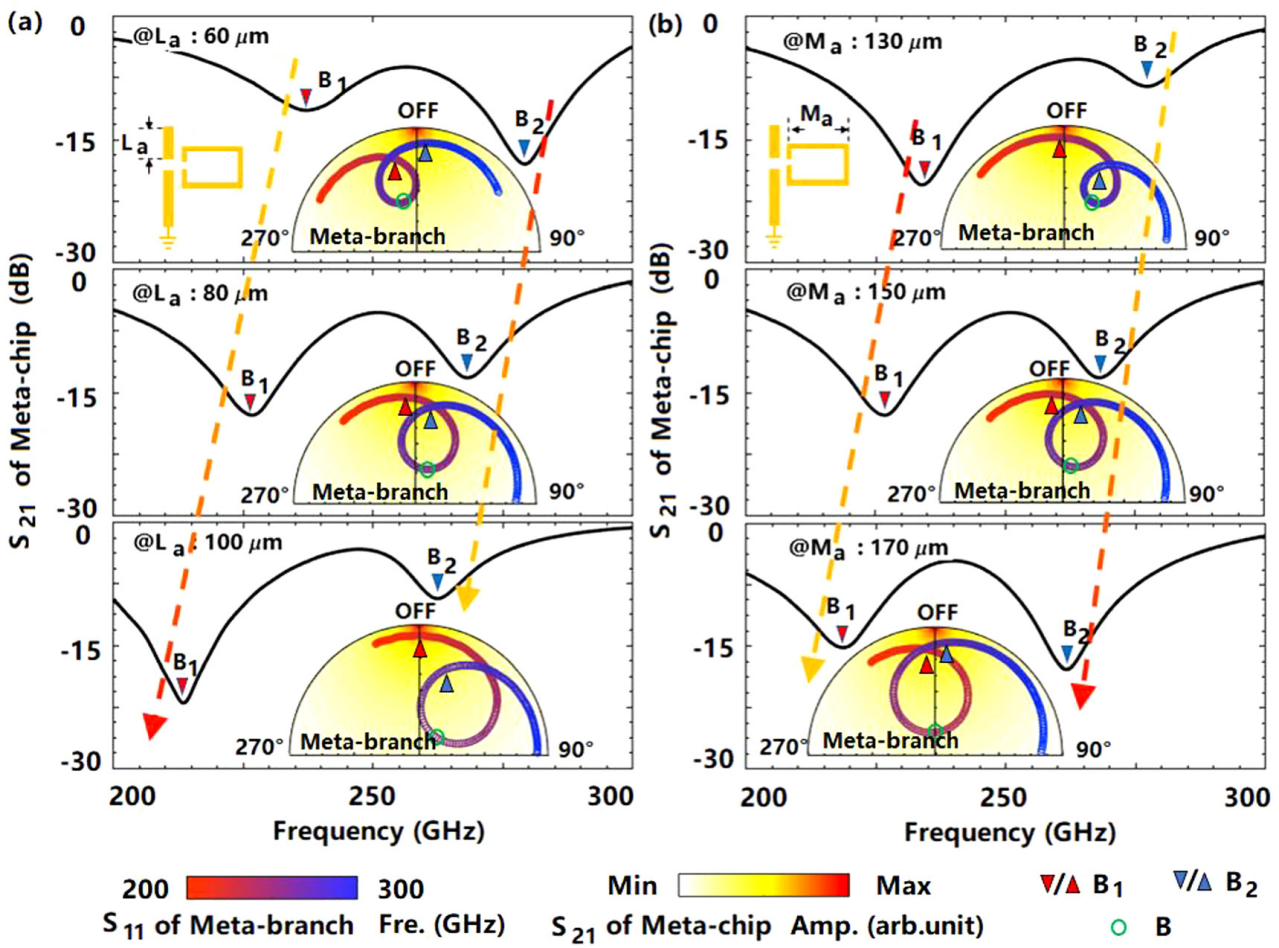
The dependences of the *S*_21_ on the size parameters of meta-branch. (a) The spectra of *S*_21_ of the meta-chip for different *L*_*a*_, the insets are the amplitude and phase characteristics of *S*_11*b*_ in the polar coordinates for the corresponding *L*_*a*_, and *L*_*a*_ is the length of the first branch; (b) The spectra of *S*_21_ of the meta-chip for different *M*_*a*_, the insets are the amplitude and phase characteristics of *S*_11*b*_ in the polar coordinates for the corresponding *M*_*a*_, and *M*_*a*_ is the length of the upper arm of the C-ring.

The revealed mechanism shows that the major factor affecting the switching characteristic of the meta-chip is the amplitude and phase characteristics of *S*_11*b*_. Taking the peak B of *S*_11*b*_ for example, Figure 4 shows the dependences of the *S*_21_ on the size parameters of meta-branch. As the insets shown in Figure 4a, the increase of *L*_*a*_ leads to the clockwise rotation of *S*_11*b*_ as a whole, and the non-resonant points are moved to the boundary of *S*_11*b*_ unit circle. This is because that the peak B mainly comes from the resonance within the C-ring. On the one hand, the increase of *L*_*a*_ directly changes the electrical length of the first branch, resulting in a phase change. On the other hand, the density of the accumulated charges is also changed owing to the varying fields distribution caused by the changing electrical length. Thus, the resonance intensity at peak B is changed, which leads a variation of *S*_11*b*_ amplitude further. Accordingly, as shown in Figure 4a, the varied amplitude and phase lead to the change of the “OFF” status of the meta-chip. It is found that the operating frequency and *S*_21_ at “OFF” status B_1_ decrease with the increase of *L*_*a*_. While for the “OFF” status B_2_, the operating frequency decreases, and *S*_21_ increases.

While for the size of the C-ring, the increase of *M*_*a*_ reduces the corresponding resonant frequency, which leads a counterclockwise movement of the resonance peaks in the insets of Figure 4b. It is found that the resulting amplitude change of *S*_11*b*_ makes the “OFF” status at B_1_ and B_2_ move gradually away from and close to the optimal switching point, respectively. Thus, as shown in Figure 4b, the increase of *M*_*a*_ reduces the frequencies of the “OFF” states at B_1_ and B_2_, and the *S*_21_ at point B_1_ and B_2_ increases and decreases, respectively. The above results further show that the switching characteristics of the meta-chip mainly depend on the amplitude and phase characteristics of meta-branches, which can be designed on demand by artificial microstructure. In this way, the device performance can be optimized in the case of strong coupling.

## 3 Fabrication and experiment

**Figure 5:**
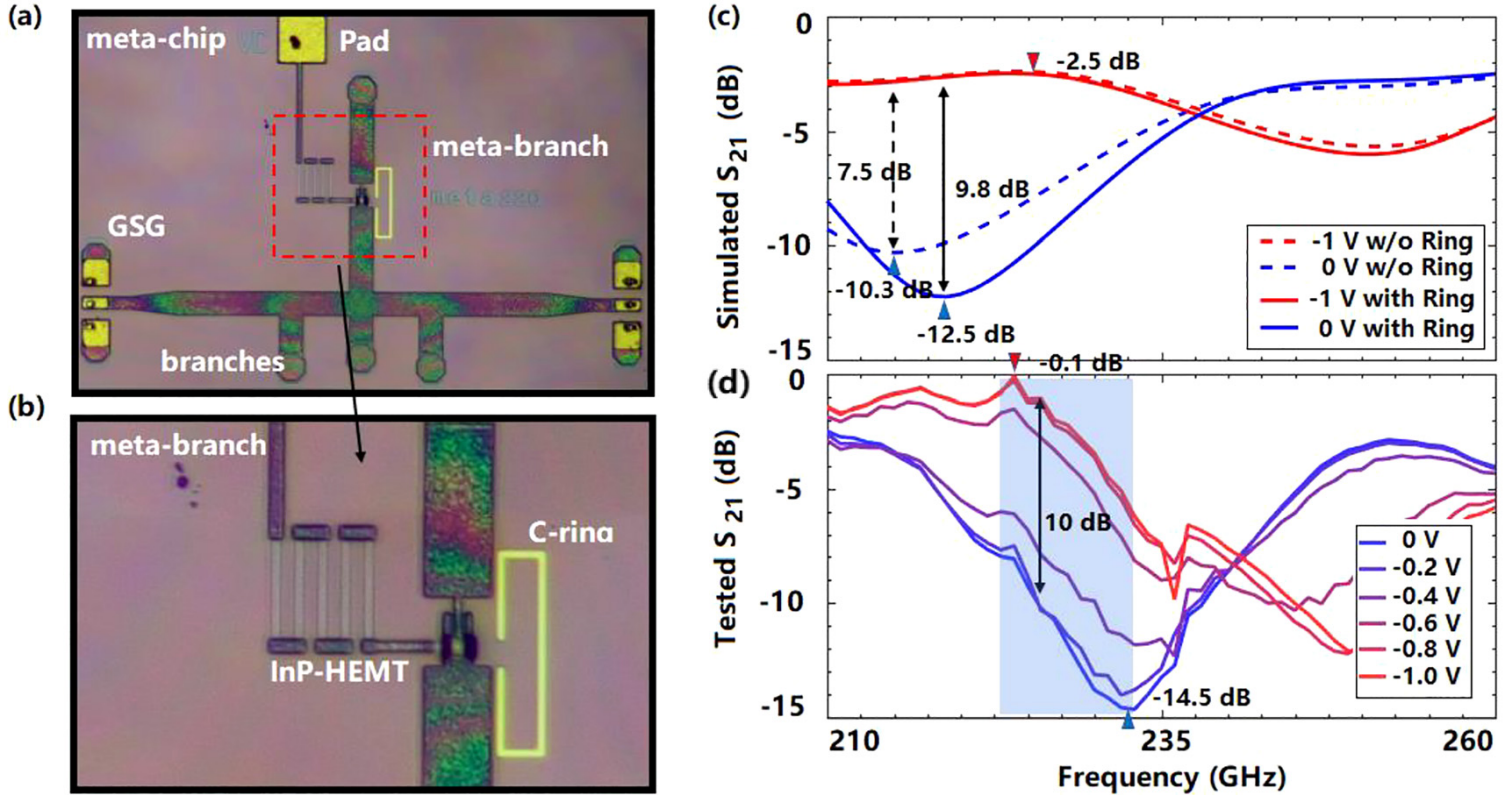
The fabricated meta-chip and the tested results. (a) The photo of fabricated meta-chip; (b) the partially enlarged view of the meta-branch; (c) the simulation results of *S*_21_ for the meta-chip and that without the ring for the 90 nm gate length InP-HEMT; (d) the tested results of *S*_21_ for the meta-chip.

Based on the revealed mechanism, a meta-chip with single InP-HEMT working at 220 GHz is designed with the 90 nm gate length processing technology, which is fabricated by using a self-aligned T-grid gate structure, multilayer photoresist, electron beam lithography, and dry etching. The thickness of the InP substrate is 70 μm, and the microstrip line width is 48 μm. The micrograph of the fabricated switch meta-chip is shown in Figure 5a, in which the meta-branch is connected parallel to the main microstrip line, three microstrip branches are designed on the main line for impedance matching, and ground-signal-ground structures are designed at the input and output port. The partially enlarged view of the meta-chip switch is shown in Figure 5b, and the C-ring is located near the InP-HEMT. The simulation and test results are demonstrated in Figure 5c and d. It is found in Figure 5c that, compared with the chip without C-ring loading, the switching ratio of meta-chip is increased by 20% while ensuring the same rather low insertion loss. This is because that the coupling between microstrip branches, InP-HEMT and C-ring modifies the impedance of the meta-branch, which meets the revealed optimal switching conditions, and enhances the “ON/OFF” efficiency of the fabricated HEMT further. The test results show that the terahertz waves are gradually switched from “ON” to “OFF” by controlling the gate voltage from −1 to 0 V within a bandwidth of 10 GHz. The minimum insertion loss of the meta-chip is less than 1 dB, and the maximum switching ratio is larger than 10 dB, which agrees with the trend of the simulation results. Further, the spectrum of the modulated terahertz signal shows an effective switching at 225 GHz with 0.5 GHz controlling signal, which indicates a switch velocity of 2 ns. It also can be found that there appears a frequency shift caused by the errors induced by processing technology, which shifts the operating frequency from 220 to 235 GHz, as shown in Figure 5c, and this can be modified by the machining iterations.

## 4 Summary

In this paper, a parallel topology meta-chip switch with C-ring loading is presented. The meta-branch is composed of the microstrip branches, InP-HEMT, and C-ring, which is designed according to the optimal switching points revealed by combing the equivalent circuit theory and electromagnetic coupling. By controlling the density of TDEG of InP-HEMT, the perturbation induced by weak coupling in the meta-branch and resonance caused by strong coupling is converted, and then the switching of the meta-chip is realized. Based on this mechanism, the optimization of the switch chip is transformed into the design of artificial microstructure in the meta-branch. Thus, the optimal switch can be realized by introducing structural schemes in metamaterials, which provides an effective idea for device design and performance optimization in sub-wavelength spatial scale.

According to this mechanism, in this paper, a parallel topology meta-chip switch with a single InP-HEMT working at 220 GHz is designed and fabricated by using the 90 nm gate length process. The test results show that the minimum insertion loss of the meta-chip is reduced to less than 1 dB, and the switching ratio is increased to 10 dB. Compared with the traditional chip without C-ring loading, the switching ratio is increased by 20% while ensuring a rather low insertion loss. In the case of meta-chip, the strong coupling in sub-wavelength scale can be controlled by introducing the artificial microstructures, and then the performances of the chip can be improved further by adding more transistors. Therefore, the meta-chip loaded by artificial microstructure provides a new idea for the chip design in the terahertz band, which is conducive to solving the problems of strong coupling and large parasitic parameters under the condition of sub-wavelength scale.

## References

[1] Ferguson B., Zhang X. C. (2002). Materials for terahertz science and technology. *Nat. Mater.*.

[2] Ji B., Han Y., Liu S., Tao F., Li C. (2021). Several key technologies for 6G: challenges and opportunities. *IEEE Commun. Stand. Mag.*.

[3] Koenig S., Lopez-Diaz D., Antes J. (2013). Wireless sub-THz communication system with high data rate. *Nat. Photonics*.

[4] Zhou T., Zhang Yaxin, Zhang Bo (2021). Terahertz direct modulation techniques for high-speed communication systems. *China Commun.*.

[5] Li X., Zhan Y., Li O. (2017). 225–255-GHz InP DHBT frequency tripler MMIC using complementary split-ring resonator. *J. Infrared, Millim. Terahertz Waves*.

[6] Ji D., Zhang B., Wang J., Yang Y., Chen X. (2020). Analysis of welding pad for terahertz hybrid integrated mixer. *IEEE Access*.

[7] Ji D., Zhang B., Yang Y., Niu Z., Chen X. (2019). A 220-GHz third-harmonic mixer based on balanced structure and hybrid transmission line. *IEEE Access*.

[8] Yamasaki S., Yasui A., Amemiya T. (2017). Optically driven terahertz wave modulator using ring-shaped microstripline with GaInAs photoconductive mesa structure. *IEEE J. Sel. Top. Quant. Electron.*.

[9] Amakawa S., Fujishima M. (2019). *300-GHz-band CMOS Transmitter and Receiver Modules with WR-3.4 Waveguide interface. 2019 IEEE MTT-S International Microwave Conference on Hardware and Systems for 5G and beyond (IMC-5G)*.

[10] Fedotov V. (2021). Phase control of terahertz waves moves on chip. *Nat. Photonics*.

[11] Yuan C. W., Yu L. Z., Zhang Q., Xu L., Zhao X. H. (2020). A novel high-power waveguide phase shifter with continuous linear phase adjustment. *Rev. Sci. Instrum.*.

[12] Thome F., Ambacher O. (2017). Highly isolating and broadband single-pole double-throw switches for millimeter-wave applications up to 330 GHz. *IEEE Trans. Microw. Theor. Tech.*.

[13] Shivan T., Hossain M., Stoppel D. (2018). 220–325 GHz high‐isolation SPDT switch in InP DHBT technology. *Electron. Lett.*.

[14] Deza J., Ouslimani A., Konczykowska A., Kasbari A., Godin J., Pailler G. (2012). 65 GHz small-signal-bandwidth switched emitter follower in InP heterojunction bipolar transistors. *Electron. Lett.*.

[15] Chowdhury A. R., Ness R., Joshi Ravi P. (2018). Assessing lock-on physics in semi-insulating GaAs and InP photoconductive switches triggered by subbandgap excitation. *IEEE Trans. Electron. Dev.*.

[16] Ajayan J., Nirmal D., Ravichandran T. (2018). InP high electron mobility transistors for submillimetre wave and terahertz frequency applications: a review. *AEU-Int. J. Elec. Commun.*.

[17] Cui T. J., Qi M. Q., Wan X., Zhao J., Cheng Q. (2014). Coding metamaterials, digital metamaterials and programmable metamaterials. *Light Sci. Appl.*.

[18] Hu T., Strikwerda A. C., Fan K., Padilla W. J., Zhang X., Averitt R. (2009). Reconfigurable terahertz metamaterials. *Phys. Rev. Lett.*.

[19] Ju L., Geng B. S., Horng J. (2011). Graphene plasmonics for tunable terahertz metamaterials. *Nat. Nanotechnol.*.

[20] Zheludev N. I., Kivshar Y. S. (2012). From metamaterials to metadevices. *Nat. Mater.*.

[21] Chen H.-T., Padilla W. J., Zide J. M, Gossard A. C., Taylor A. J., Averitt R. D. (2006). Active terahertz metamaterials devices. *Nature*.

[22] Gu J., Singh R., Liu X. J. (2012). Active control of electromagnetically induced transparency analogue in terahertz metamaterials. *Nat. Commun.*.

[23] Zhang S., Zhou J. F., Park Y. S. (2012). Photoninduced handedness switching in terahertz chiral metamolecules. *Nat. Commun.*.

[24] Zhang C., Zhou G. C., Wu J. B., Tang Y., Wu P. H. Active control of terahertz waves using vanadium-dioxide-embedded metamaterials. *Phys. Rev. Appl.*.

[25] Han S., Cong L. Q., Srivastava Y. K (2019). All-dielectric active terahertz photonics driven by bound states in the continuum. *Adv. Mater.*.

[26] Buchnev O., Nina P., Malgosia K., Nikolay I. Z., Vassili A. F. (2015). Electrically controlled nanostructured metasurface loaded with liquid crystal: toward multifunctional photonic switch. *Adv. Opt. Mater.*.

[27] Zhao Y. T., Wu B., Huang B. J., Cheng Q. (2017). Switchable broadband terahertz absorber/reflector enabled by hybrid graphene-gold metasurface. *Opt. Express*.

[28] Xu H., Bi K., Hao Y. (2018). Switchable complementary diamond-ring-shaped metasurface for radome application. *IEEE Antenn. Wireless Propag. Lett.*.

[29] Lei L., Lou F., Tao K., Huang H., Cheng X., Xu P. (2019). Tunable and scalable broadband metamaterial absorber involving **vo**
_
**2**
_-based phase transition. *Photon. Res.*.

[30] Chen A., Song Z. Y. (2019). Wideband polarization-insensitive dielectric switch for mid-infrared waves realized by phase change material Ge3Sb2Te6. *EPL*.

[31] Choi C., Lee S. Y., Mun S. E (2019). Metasurface with nanostructured Ge2Sb2Te5 as a platform for broadband‐operating wavefront switch. *Adv. Opt. Mater.*.

[32] Srivastava Y. K., Manjappa M., Cong L. (2018). A superconducting dual‐channel photonic switch. *Adv. Mater.*.

[33] Pham T. S., Bui H. N., Lee J. W. (2019). Wave propagation control and switching for wireless power transfer using tunable 2-D magnetic metamaterials. *J. Magn. Magn Mater.*.

[34] Xu S. T., Fan F., Ji Y. Y., Cheng J. R., Chang S. J. (2019). Terahertz resonance switch induced in the polarization conversion of liquid crystal in compound metasurface. *Opt. Lett.*.

[35] Su X., Ouyang C., Xu N. (2015). Broadband terahertz transparency in a switchable metasurface. *IEEE Photonics J.*.

